# Rapid evaluation of heterologous chimeric RBD-dimer mRNA vaccine for currently-epidemic Omicron sub-variants as booster shot after inactivated vaccine

**DOI:** 10.1016/j.bsheal.2023.02.002

**Published:** 2023-03-02

**Authors:** Qian Chen, Pei Du, Yuxuan Han, Xuehui Ma, Rong Zhang, Xiaoyu Rong, Xu Zhao, Renyi Ma, Huiting Yang, Anqi Zheng, Qingrui Huang, Jinghua Yan, Hui Wang, Xin Zhao, Lianpan Dai, George F. Gao, Qihui Wang

**Affiliations:** a Institutes of Physical Science and Information Technology, Anhui University, Hefei 230601, China; b CAS Key Laboratory of Pathogen Microbiology and Immunology, Institute of Microbiology, Chinese Academy of Sciences (CAS), Beijing 100101, China; c Savaid Medical School, University of Chinese Academy of Sciences, Beijing 100049, China; d School of Laboratory Medicine and Life Sciences, Wenzhou Medical University, Wenzhou 325035, China; e Beijing Institute of Biological Products Company Limited, Beijing 100176, China

**Keywords:** SARS-CoV-2, RBD-dimer, mRNA Vaccine, Broad-spectrum, Omicron, BA.5

## Abstract

With continuous mutations of severe acute respiratory syndrome coronavirus 2 (SARS-CoV-2), the severe immune escape of Omicron sub-variants urges the development of next-generation broad-spectrum vaccines, especially as booster jabs after high-level vaccination coverage of inactivated vaccines in China and many other countries. Previously, we developed a coronavirus disease 2019 (COVID-19) protein subunit vaccine ZF2001® based on the tandem homo-prototype receptor-binding domain (RBD)-dimer of the SARS-CoV-2 spike protein. We upgraded the antigen into a hetero-chimeric prototype (PT)-Beta or Delta-BA.1 RBD-dimer to broaden the cross-protection efficacy and prove its efficiency with protein subunit and mRNA vaccine platforms. Herein, we further explored the hetero-chimeric RBD-dimer mRNA vaccines and evaluated their broad-spectrum activities as booster jabs following two doses of inactivated vaccine (IV) in mice. Our data demonstrated that the chimeric vaccines significantly boosted neutralizing antibody levels and specific T-cell responses against the variants, and PT-Beta was superior to Delta-BA.1 RBD as a booster in mice, shedding light on the antigen design for the next-generation COVID-19 vaccines.

## Highlights

• **Scientific questions:** The continuous mutation of SARS-CoV-2 leads to severe escape that gradually renders the vaccines based on prototype SARS-CoV-2 ineffective, which urges the development of a new generation of broad-spectrum vaccines for boosting vaccination.
• **Evidence before this study:** Previously, we developed the COVID-19 protein subunit vaccine ZF2001® based on the RBD-dimer of prototype SARS-CoV-2, which has been approved for use in China, Uzbekistan, Indonesia and Columbia. To broaden the cross-protection efficacy, we upgraded the antigen into a hetero-chimeric prototype (PT)-Beta or Delta-BA.1 RBD-dimer, and proved its efficiency with protein subunit and mRNA vaccine platforms.
• **New findings:** In this study, the designs of hetero-chimeric RBD-dimer mRNA vaccines were further explored, and their broad-spectrum activities as booster jabs following two doses of inactivated vaccine in mice were evaluated. The results demonstrated that the chimeric vaccines greatly boosted neutralizing antibody levels and specific T-cell responses against the variants, and PT-Beta was superior to Delta-BA.1 RBD as a booster in mice.
• **Significance of the study:** These results provide basis for choosing effective antigens for booster jabs.

## 1 Introduction

It has been over three years since the start of the coronavirus disease 2019 (COVID-19) pandemic caused by severe acute respiratory syndrome coronavirus 2 (SARS-CoV-2), resulting in a death toll of over 7 million cases and countless economic losses (https://www.who.int). To prevent the disease, saturated vaccine Research & Development strategies have been explored from the very beginning [1], [2], [3], [4], with mRNA vaccines [5], [6], [7], [8], inactivated virus vaccines [9], [10], protein subunit vaccines [11], adenoviral vector vaccines [12], [13] and DNA vaccines [14]. Currently, large-scale vaccination campaigns have been implemented in many countries and have shown protection against COVID-19, especially for severe disease and death [4]. However, new SARS-CoV-2 variants continue to emerge and circulate; some variants showed resistance to the vaccine-induced immune responses, such as variants of concern (VOCs) Beta, Gamma, and Omicron. Accompanying the waning of anti-viral immunity, which leads to breakthrough infections, boost vaccination was necessary to enhance the immune responses and protective efficacy.

The global dominant circulating SARS-CoV-2 strains sequentially changed rapidly in the past year from Delta to Omicron. Therefore, to fight against the surging waves of SARS-CoV-2 variants, especially the currently circulating Omicron sub-variants, developing next-generation vaccines that could induce broad-spectrum immune responses is urgently needed, especially as booster jab vaccines.

Previously, we developed the COVID-19 protein subunit vaccine ZF2001® based on the tandem homo-prototype receptor-binding domain (RBD)-dimer of SARS-CoV-2 spike (S) protein [11], [15], which has been approved for use in China, Uzbekistan, Indonesia, and Columbia [16], [17]. Notably, the feasibility of this tandem RBD-dimer design allows the combination of variant RBDs and broadens their cross-protection efficiency. Thus, to confront the emerging SARS-CoV-2 variants, we further designed the protein subunit vaccine using hetero-chimeric RBD-dimer as the immunogen and demonstrated their capability to induce broader and stronger protective immune responses against VOCs than the homotypic RBD-dimers [18]. Furthermore, by applying the mRNA vaccine platform, this design was further proved to induce robust humoral and cellular immune responses and conferred protection against the SARS-CoV-2 challenge [7], [19].

In this work, we expanded the combinations of the RBD dimers by constructing a panel of seven mRNA vaccines using RBDs from the same or different variants of SARS-CoV-2 (prototype, Beta, Delta, and Omicron BA.1) and systematically investigated their immunogenicity in naïve mice. Moreover, we evaluated the induction of immune responses by chimeric RBD-dimer mRNA vaccine candidates as boosters in mice prime-vaccinated with COVID-19-inactivated vaccines (IV) widely used in China and many other countries. These results provided a swift evaluation platform of broad-spectrum mRNA vaccine candidates for next-generation COVID-19 vaccines and will significantly contribute to the global efforts to fight against COVID-19.

## 2 Materials and methods

### 2.1 Plasmids, cells and animals

The human-codon optimized DNA sequence of SARS-CoV-2 RBDs (prototype: EPI_ISL_402119, Delta: EPI_ISL_2378732, Beta: EPI_ISL_678597, BA.1: EPI_ISL_6640916) were synthesized by Genscript and subcloned into the vector for *in vitro* mRNA transcription. HEK293T cells (ATCC CRL-3216) and Vero cells (ATCC CCL81) were cultured in Dulbecco’s modified Eagle’s medium (DMEM) supplemented with 10% fetal bovine serum (FBS) at 37 °C. BALB/c mice (female, 6–8 weeks) were purchased from Beijing Vital River Animal Technology Co., Ltd (licensed by Charles River) and were housed in a specific-pathogen-free (SPF) mouse facilities in IMCAS with temperature-, humidity- and light cycle-control (20 ± 2 °C; 50% ± 10%; light, 7:00–19:00; dark, 19:00–7:00).

### 2.2 mRNA *in vitro* transcription and 5′-capping

mRNA was transcribed *in vitro* using T7 high yield RNA transcription kit (Novoprotein) on linearized plasmids encoding dimeric RBDs. A 104 nucleotide-long poly(A) tail was transcribed into mRNA. UTP was replaced by 1-methylpseudourine-5′-triphosphate during *in vitro* transcription to generate nucleotide-modified mRNA. 5′-Capping was conducted using the Cap 1 capping system (Novoprotein). mRNA was purified by precipitation with LiCl at −20 °C overnight, centrifugation at 18,800 × g for 20 min at 4 °C, and resuspension with RNase-free water. Purified mRNA was verified by agarose gel electrophoresis and stored at −80 °C until use.

### 2.3 Lipid nanoparticle (LNP) encapsulation of mRNA

LNP encapsulation was conducted using the Nanoassemblr Benchtop platform (Precision Nanosystems). Basically, mRNA diluted in the aqueous solution (pH = 4.0) was quickly mixed with lipids diluted in ethanol in a microfluidics system, which leads to the encapsulation of mRNA by lipids in a self-assembly process. LNPs used in this study were assembled by an ionizable cationic lipid, phosphatidylcholine, cholesterol, and PEG-lipid at a ratio of 50:10:38.5:1.5 (mol/mol). The mRNA to lipid ratio was approximately 0.05 (wt/wt). LNP-encapsulated mRNAs were quantified using Quan-iT Ribogreen RNA reagent (Thermo Fisher). LNPs were stored at 4 °C at a concentration of RNA of around 0.5 mg/ml.

### 2.4 Particle size, zeta potential and cryo-electron microscopy of LNPs

For determining the particle sizes and zeta potential of LNPs, LNPs were diluted into deionized water at pH 4.0 or pH 7.4. Diluted LNPs were added into folded capillary zeta cells and loaded into Zetasizer Pro (Malvern Panalytical). Particle size distribution and polydispersity index were measured with dynamic light scattering. Zeta potential was measured by Zeta. For cryo-electron microscopy, LNPs were transferred onto a glow-discharged ultrathin carbon-coated copper grid, blotted for 2 s with filter paper in FEI Vitrobot Mark IV (Thermo Fisher), followed by quick plugging into liquid ethane. Frozen grids were loaded into a Talos transmission electron microscope (Thermo Fisher Scientific) equipped with a field emission gun operated at 200 kV. Images were recorded on a direct electron detector (ED20).

### 2.5 mRNA transfection and western blot

HEK293T cells were transfected with TransIT-mRNA kit (Mirus Bio). Basically, mRNA (1 μg) was added to 100 μl serum-free Opti-MEM together with TransIT-mRNA reagent (2 μl) and booster reagent (2 μl). The complex was incubated for 3 min before being added dropwise to 5 × 10^5^ cells cultured in complete medium in 12 well plates. Supernatants were collected 36 h post-transfection and stored at −20 °C until use. For western blot, supernatant samples were combined with loading buffer with dithiothreitol, separated by 10% SDS-PAGE and transferred to PVDF membrane using a semi-dry apparatus (WIX Technology). Then, the membrane was blocked with 5% non-fat milk diluted in TBS-T buffer, blotted with SARS-CoV-2 Spike/RBD primary antibody (Sino Biological) for 1 h and goat anti-rabbit IgG-HRP secondary antibody (Easybio) for 1 h. Finally, the membrane was developed using Beyotime BeyoECL Plus (Beyotime Biotech).

### 2.6 Animal experiments

All vaccines were immunized by injecting female BALB/c mice aged 6–8 weeks via the i.m. route.

For evaluating humoral immunogenicity of mRNA vaccines, groups of mice (n = 5) were immunized with two doses of mRNA vaccine (5 μg/mouse per dose) or empty LNP as placebo control on day 0 and day 14. Blood samples were collected by retro-orbital blood collection method on day 14 and by cardiac puncture on day 28. Blood samples were further centrifuged and the serum in supernatants was stored at −80 °C until use.

For evaluating the cellular immunogenicity of mRNA vaccines, groups of mice (n = 6) were immunized with two doses of mRNA vaccine (5 μg/mouse per dose) or empty LNP as a placebo control on day 0 and day 14. Mice were sacrificed 7 days post the second immunization on day 21 and spleens were collected immediately after sacrifice. Then, spleens were homogenized with a tissue grinder in 1 ml of serum-free DMEM, filtered with a 40 μm cell strainer (Corning), followed by lysis of red blood cells with red blood cell lysis buffer (Solarbio Life Science). Splenocytes were stained with 0.4% trypan blue solution and counted using Celldrop FL automated cell counter (DeNovix). Live splenocytes were then immediately used for intracellular cytokine staining (ICS) assay and ELISpot assay.

For evaluating the immunogenicity of booster vaccination, groups of mice were immunized with two doses of inactivated vaccine (2.6U/mouse per dose) or Al adjuvant as placebo control on day 0 and day 21, followed by a third injection of mRNA vaccine (10 μg/mouse) or inactivated vaccine (2.6U/mouse) on day 35. The group injected with two doses of Al was further injected with empty LNP or Al as the placebo control for mRNA vaccines or inactivated vaccines (BBIBP-CorV, Sinopharm), respectively. On day 35, blood samples were collected by retro-orbital blood collection method. On day 49, mice were sacrificed. Blood samples and spleens were collected immediately after the sacrifice. Blood samples and spleens were further processed with the similar protocol mentioned above.

### 2.7 Enzyme-linked immunosorbent assay (ELISA)

ELISA was performed with the method mentioned in our previous work [20]. Briefly, ELISA plates (Corning) were coated overnight with the RBD of SARS-CoV-2 prototype, Delta, Beta, BA.1, BA.1.1, BA.2, BA.2.12.1, BA.3 or BA.5 recombinant protein (0.2 μg/ml) 0.05 M carbonate-bicarbonate buffer, pH 9.6. Then, the plates were blocked in 5% non-fat milk diluted in PBS-T buffer. Serum samples were subjected to a threefold serial dilution starting from 1:200 or 1:1,000. After adding the diluted serum to each well, the plates were incubated for 1 h at 37 °C. Goat anti-mouse IgG-HRP antibody was added to plates as a secondary antibody and incubated for 1 h at 37 °C. The plates were developed with 3,3′,5,5′-tetramethylbenzidine (TMB) substrate. After stopping the reaction with 2 M hydrochloric acid, the absorbances at 450 nm and 630 nm were measured using a microplate reader (PerkinElmer). Absorbance values were calculated by subtracting the absorbance at 630 nm from that at 450 nm of the same well. Endpoint titers were defined as the highest reciprocal dilution of serum to yield an absorbance greater than 2.1-fold of the background values. Antibody titer below the limit of detection was determined as one-third of the detection limit.

### 2.8 Pseudovirus neutralization assay

The construction of VSV-backbone pseudotyped viruses of SARS-CoV-2 variants and neutralization assay were performed as previously described [20]. Briefly, the codon-optimized spike protein of SARS-CoV-2 variants with 18C-terminus amino acid truncation was constructed into the pCAGGS vector. HEK293T cells were transfected with 30 μg of each pCAGGS vector. After 24 h, VSV-ΔG-G-GFP pseudotyped virus was added into cell culture and removed after 1 h incubation. Then, the cell culture medium was changed into a fresh complete DMEM medium with the anti-VSV-G antibody (I1-Hybridoma ATCC® CRL2700™). Supernatants were collected after 30 h incubation and filtered by a 0.45 μm sterile filter, aliquoted for single use, and stored at −80 °C. For neutralization assay, the heat-inactivated (56 °C, 30 min) serum samples were subjected to a twofold serial dilution started from 1:20 or 1:80. Each pseudotyped virus was mixed with an equal volume of serially diluted serum and incubated at 37 °C for 1 h, and then 100 μl mixture was added onto pre-plated Vero cells in 96 well plates. After 15 h incubation, the transducing units (TU) numbers were calculated on a CQ1 confocal image cytometer (Yokogawa).

### 2.9 ICS assay

For the ICS assay, fresh mouse splenocytes were added to 96 well plates (1 × 10^6^ cells/well). Cells were stimulated with each peptide pool (2 μg/ml for each peptide) for 3 h, and incubated with Golgiplug (BD Biosciences) for 6 h at 37 °C. Then, cells were harvested, blocked with recombinant CD16/32 protein, and stained with PE/Cy7 anti-CD3, APC/Cy7 anti-CD4 and PerCP/Cy5.5 anti-CD8α antibodies (Biolegend). After fixation and permeabilization by permeabilizing buffer (BD Biosciences), cells were further stained with PE anti-IFN-γ antibody (Biolegend). Flow cytometric analysis was conducted using a BD LSRFortessa flow cytometer (BD Biosciences) with a high-throughput system. Data were analyzed using FlowJo 10.0 (Fig. S1).

### 2.10 Enzyme-linked immunospot (ELISpot) assay

For ELISpot assay, flat-bottom 96 well plates were pre-coated with 10 g/ml anti-mouse IFN-γ Ab (BD Biosciences) overnight at 4 °C and blocked for 2 h at room temperature. Fresh mouse splenocytes were added into the pre-coated 96 well plates (3 × 10^5^ cells/well) and stimulated with each peptide pool (2 μg/ml for each peptide) for 20 h. Negative control wells were not stimulated with the peptide pool. Phytohemagglutinin (PMA) was added to positive control wells. After stimulation, cells were removed from plates and the plates were probed with biotinylated IFN-γ antibody, streptavidin-HRP conjugate antibody and substrate. The development was stopped by thoroughly rinsing samples with deionized water when spots became visually observable. Finally, the number of spots were determined using an automatic ELISpot reader and image analysis software (Immuno Capture 6.5.0).

### 2.11 Statistical analysis

For ELISA and neutralization assay, data are presented as geometric mean ±95% confidence interval (CI). For ICS assay, data are presented as mean ± standard error of the mean (SEM). Statistical analysis like Wilcoxon matched-pairs signed rank test, the Ordinary two-way ANOVA test, and the Mann-Whitney test were performed. All graphs and statistical analyses were generated with GraphPad Prism version 9.0.

## 3 Results

### 3.1 Design and characterization of seven mRNA vaccines with RBD-dimers of SARS-CoV-2

**Fig. 1:**
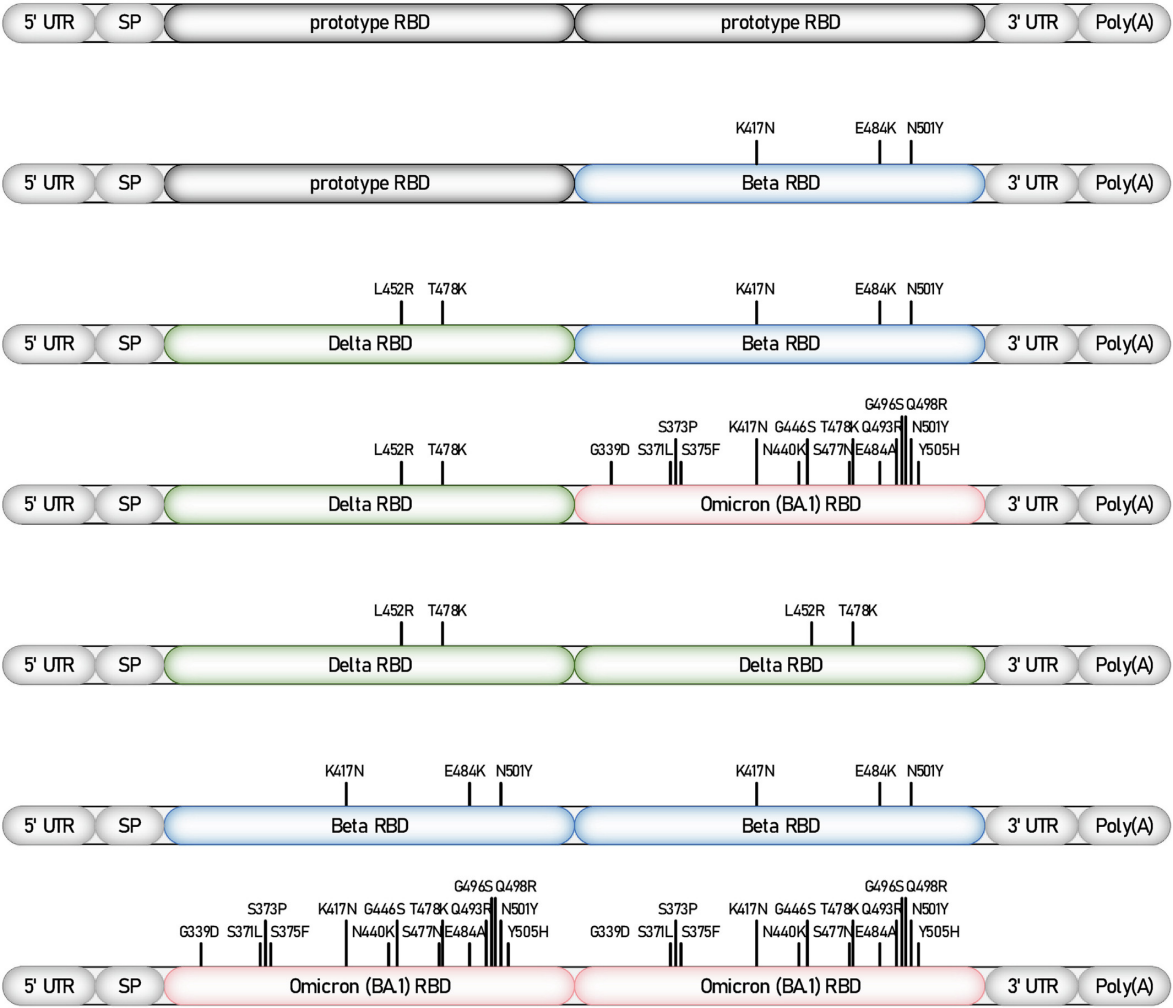
Schematic of seven mRNA vaccines with dimeric receptor-binding domains (RBDs) of severe acute respiratory syndrome coronavirus 2 (SARS-CoV-2). Seven mRNA vaccines were designed by combining the RBD of prototype, Beta, Delta and Omicron (BA.1) variants.

To systematically construct mRNA vaccines with dimeric RBDs with broad-spectrum immunogenicity, we chose RBDs from the prototype (WH-01), Beta (B.1.351), Delta (B.1.617.2), and Omicron (BA.1) variants of SARS-CoV-2 as immunogens. As a result, seven mRNA vaccines were constructed, including four having RBDs from the same variant (prototype-prototype, Delta-Delta, Beta-Beta, and Omicron-Omicron, hereafter referred to as PP, DD, BB, and OO, respectively) and three having chimeric RBDs from different variants (prototype-Beta, Delta-Beta, and Delta-Omicron; hereafter referred to as PB, DB, and DO, respectively) (Fig. 1).

**Fig. 2:**
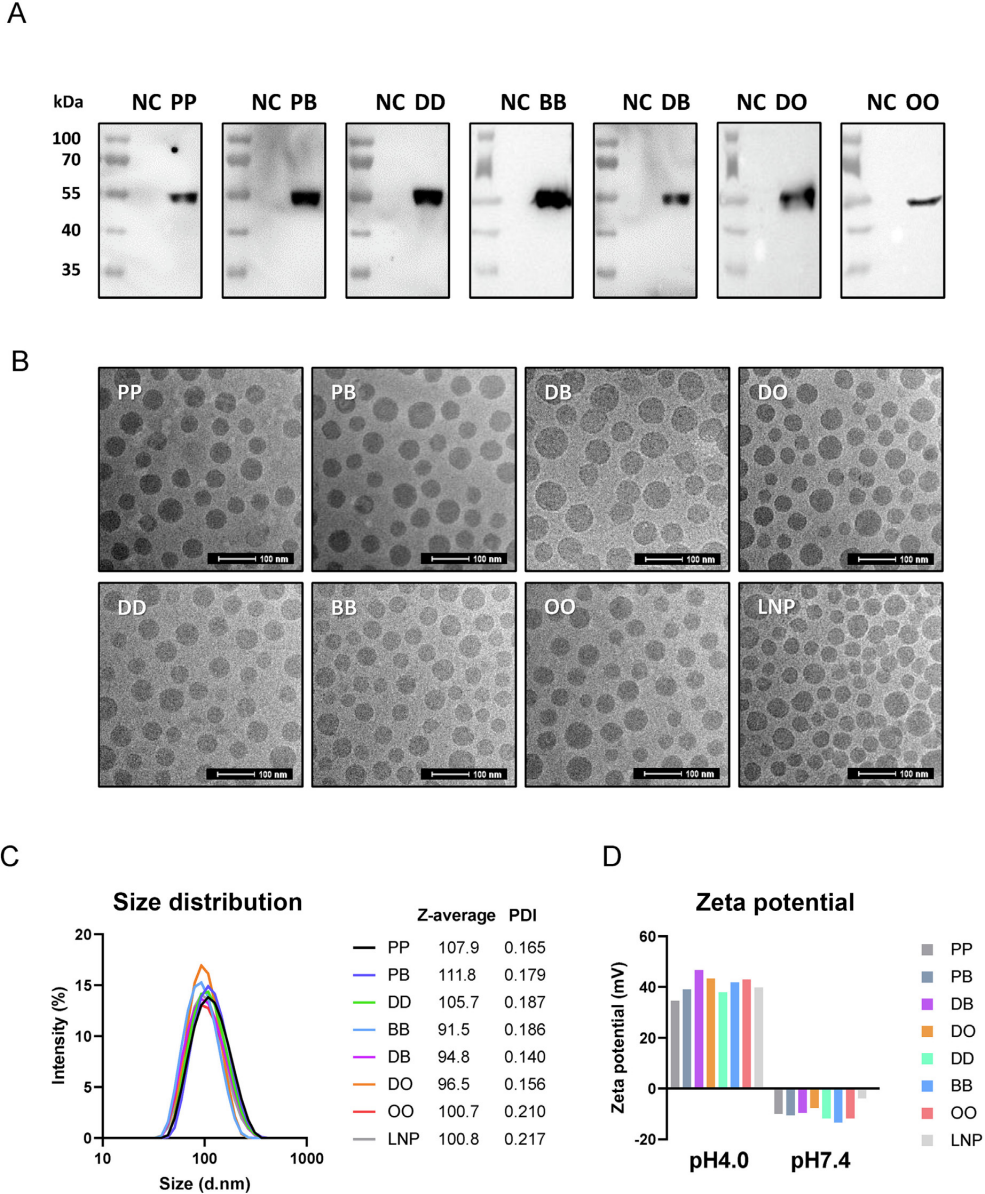
*In vitro* characterization of seven mRNA vaccines with dimeric receptor-binding domains (RBDs) of severe acute respiratory syndrome coronavirus 2 (SARS-CoV-2). A) *In vitro* expression of the seven mRNAs encoding dimeric RBDs. Each mRNA was transfected into HEK293T cells. The expression of dimeric RBDs in the supernatant was analyzed by western blot. B) Cryo-electron microscopy image of the lipid nanoparticles (LNPs) of seven mRNA vaccines and empty LNP. Scale bar, 100 nm. C) Particle size distributions of LNPs characterized by dynamic light scattering (DLS). The number in the legend indicates z-average or polydispersity index (PDI). D) Zeta potential for LNPs at pH 4.0 and 7.4. For C and D, one representative result from two independent experiments is shown.

Then, seven mRNA vaccines were prepared through *in vitro* transcription, 5′-capping, and encapsulation by (LNPs). During the *in vitro* transcription, the modified nucleoside N_1_-methylpseudouridine was used to prevent excessive innate immune responses [21]. After 5′-capping, each mRNA’s *in vitro* expression level was determined by transfecting HEK293T cells. Our results demonstrated that all dimeric RBDs were expressed at approximately 51 kD, consistent with their theoretical sizes (Fig. 2A). The LNPs were visualized using cryo-electron microscopy (cryo-EM). They displayed spherical shapes with an electron-dense core (Fig. 2B), characterizing the quality of the LNP encapsulation. Moreover, we examined the size distribution and zeta potential of the LNPs with dynamic light scattering (DLS). Our data showed that, for the LNPs of each vaccine, the z-average in phosphate-buffered saline (PBS) was within the range of 91–112 nm with the polydispersity index (PDI) from 0.140 to 0.217 (Fig. 2C), and the zeta potential increased from a negative charge at pH 7.4 to a positive charge at pH 4.0, indicating that the LNPs could be efficiently delivered into the cells (Fig. 2D).

### 3.2 Evaluation of the binding and neutralizing spectra of IgG elicited by seven mRNA vaccines

**Fig. 3:**
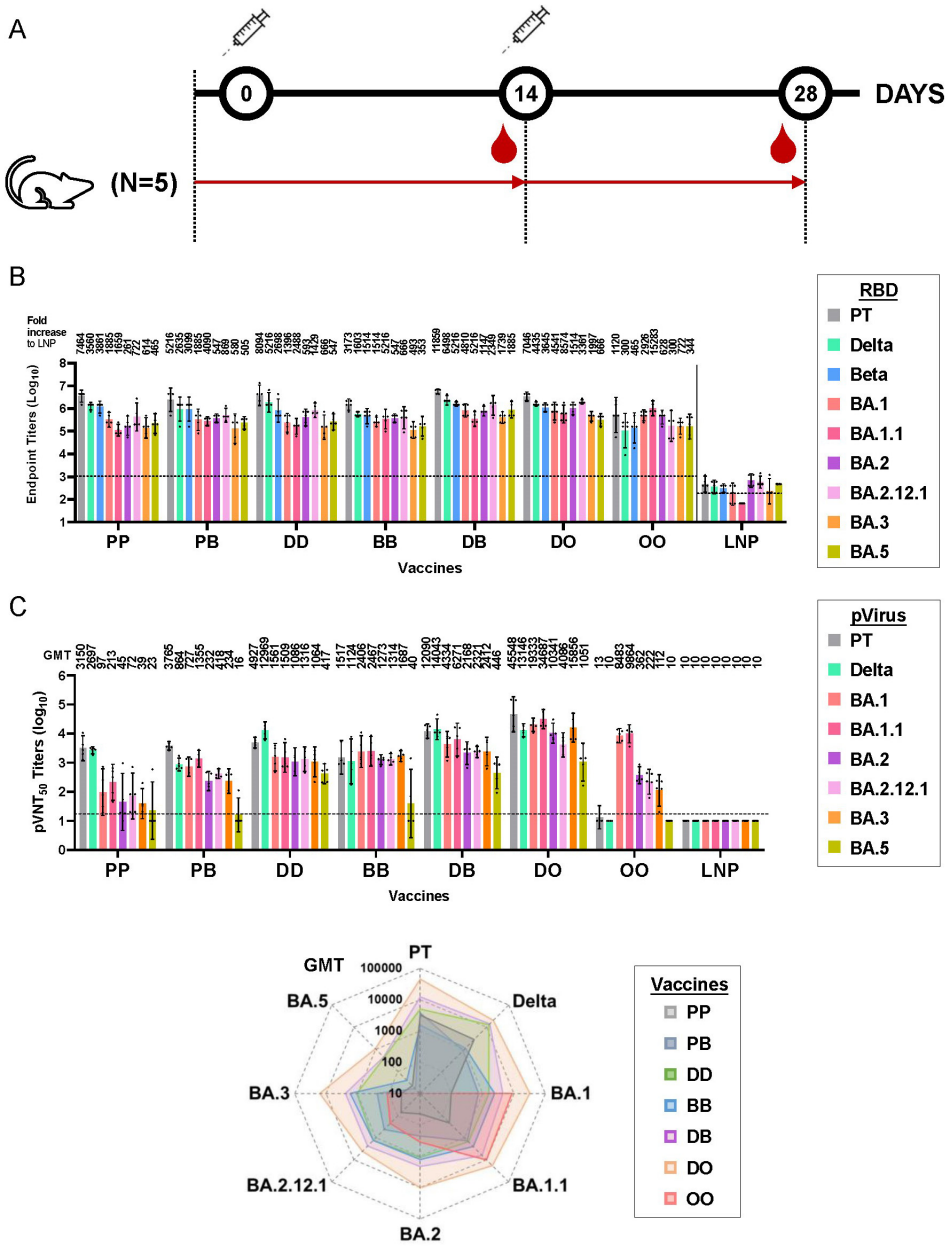
Evaluation of humoral immunogenicity of the seven mRNA vaccines with dimeric receptor-binding domains (RBDs) of severe acute respiratory syndrome coronavirus 2 (SARS-CoV-2). A) Mice immunization and sample collection schedule. B) Titers of IgG specific to the RBD of the indicated SARS-CoV-2 variants on day 28. The numbers on top indicate the fold-increase relative to the cognate RBDs in the lipid nanoparticle (LNP) group. C) NT_50_ of neutralizing antibody against the pseudotyped viruses (pVirus) of indicated SARS-CoV-2 variants. The numbers on top indicate geometric mean titer (GMT). A radar map was drawn based on GMT. All data are shown as GMT ± 95% confidential interval (CI). Pseudovirus neutralization assays were repeated twice.

We first evaluated the humoral immunogenicity of each of the seven vaccines in mice, including the IgG titers and neutralization of the pseudotyped viruses. Groups of BALB/c mice (n = 5) were immunized with two doses (days 0 and 14) of each vaccine (5 μg/mouse for each dose) or empty LNP. Serum samples were collected 14 days post each immunization (days 14 and 28) (Fig. 3A).

Using enzyme-linked immunosorbent assay (ELISA), we examined the endpoint titers of IgG specific to the RBDs of multiple SARS-CoV-2 variants (prototype, Beta, Delta, BA.1, BA.1.1, BA.2, BA.2.12.1, BA.3 and BA.5) in sera from all groups. BA.5 represents both BA.4 and BA.5 variants as it shares an identical amino acid sequence in RBD with BA.4. The results showed that all seven vaccines could elicit broad-spectrum antibodies capable of binding to the RBDs of these SARS-CoV-2 variants after two doses of vaccination. By day 28, the IgG titers specific to all examined RBDs elicited by all seven vaccines were approximately three to five orders of magnitude higher than that elicited by empty LNP (Fig. 3B and S2). Notably, among all seven vaccines, DO elicit the highest level of antibodies with the broadest binding spectra, while the IgG elicited by the OO vaccine tended to bind the RBD of BA.1 and the closely related BA.1.1 more than other variants (Fig. 3B and S2).

Using the serum samples collected on day 28, we also examined the neutralizing antibodies against eight pseudotyped viruses of SARS-CoV-2 variants, including the prototype, Delta, BA.1, BA.1.1, BA.2, BA.2.12.1, BA.3 and BA.5. The 50% neutralization titers (NT_50_) of the neutralization assays showed that the serum from each vaccinated group contained high levels of neutralizing antibodies (Fig. 3C and S3). As expected, for all seven vaccines, the neutralization spectra were similar to their corresponding binding spectra revealed by the IgG titers, as demonstrated by the radar map (Fig. 3C). Notably, DO, among the seven vaccines, exhibited the broadest neutralization spectrum, with the highest levels of neutralizing antibodies against all eight pseudotyped viruses, including prototype (45,548, hereafter referred to as geometric mean titer, GMT), Delta (13,146), BA.1 (19,333), BA.1.1 (34,687), BA.2 (10,341), BA.2.12.1 (4,086), BA.3 (15,856) and BA.5 (1,051), which was consistent with the optimal titers and broad binding spectra of IgG elicited by DO (Fig. 3C). In comparison, the neutralizing antibodies elicited by the OO vaccine were only highly effective against the pseudotyped virus of BA.1 (8,483) and BA.1.1 (9,864) but became much less effective against BA.2 (362), BA.2.12.1 (222), BA.3 (112), and showed no neutralization against prototype (13), Delta (10) or BA.5 (10). Notably, the PP vaccine elicited much lower levels of neutralizing antibodies against Omicron sub-variants (BA.1: 97; BA.1.1: 213; BA.2: 45; BA.2.12.1: 72; BA.3: 39; BA.5: 23) than that against prototype (3,510) and Delta (2,697), which highlights the necessity of updating first-generation vaccines (Fig. 3C). Interestingly, vaccines with Delta or Beta RBD as immunogens (*i.e*., PB, DD, BB, DB) demonstrated immunogenicity to Omicron. With one copy of Beta RBD, PB elicited higher levels of neutralizing antibodies against pseudotyped viruses of Omicron sub-variants, while DD and BB, with two copies of Delta or Beta RBD as immunogens, elicited further improved Omicron-specific neutralizing antibodies than PB (Fig. 3C). As a combination of Delta and Beta RBD, the DB vaccine elicited the second broadest neutralization spectra, with neutralizing antibody against prototype (12,090), Delta (14,043), BA.1 (4,334), BA.1.1 (6,271), BA.2 (2,168), BA.2.12.1 (2,321), BA.3 (2,412) and BA.5 (446), suggesting the immunogenicity of Delta and Beta RBDs against Omicron and further confirmed the advantage of chimeric RBD design (Fig. 3C). Together, these results verified that the DO vaccine induced the broadest neutralization spectra in naïve mice.

### 3.3 Evaluation of cellular immunogenicity of the seven mRNA vaccines

**Fig. 4:**
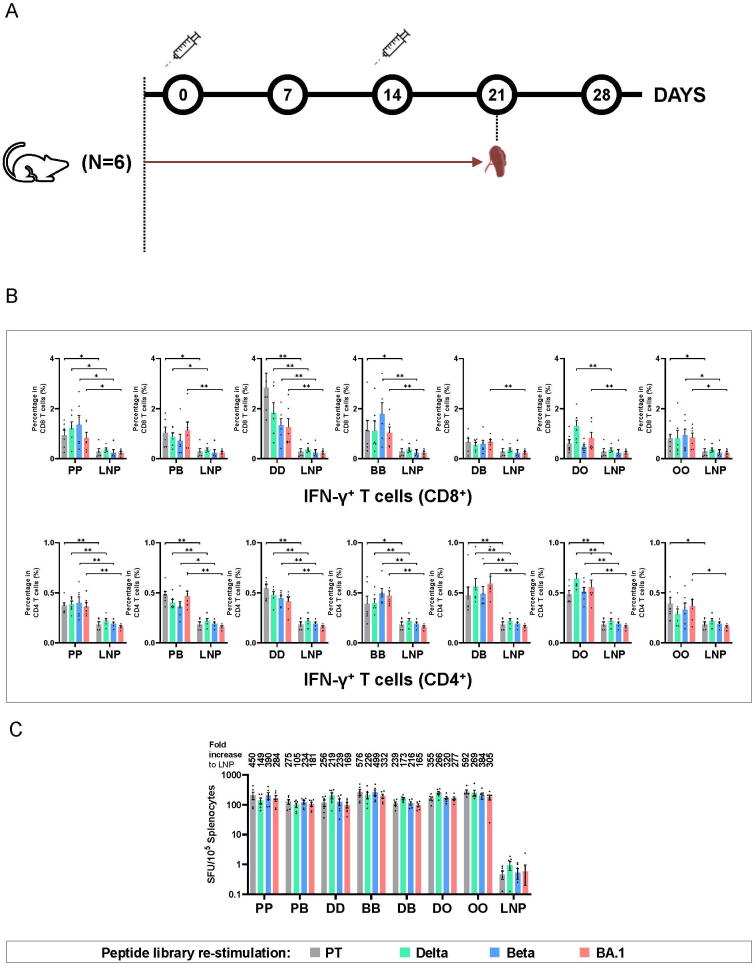
Evaluation of cellular immunity of the seven mRNA vaccines. A) Mice immunization and sample collection schedule for characterizing cellular immunity. Groups of BALB/c mice were immunized twice on days 0 and 14, splenocytes were collected on day 21. B) Intracellular cytokine staining (ICS) assays quantifying the proportions of IFNγ-secreting CD8^+^ (top) and CD4^+^ (bottom) T cells stimulated by the indicated peptide pool. C) Enzyme-linked immunospot (ELISpot) assay quantifying the IFNγ-secreting splenocytes after re-stimulation. Data are shown as means ± SEM (standard error of the mean). Statistical significances were calculated by Mann-Whitney test (*, *P* < 0.05; **, *P* < 0.01).

In addition to humoral immunogenicity, the ability to induce a cellular immune response is also an essential feature of a vaccine, particularly for mRNA vaccines known to have the advantage of cellular immunogenicity [22]. Therefore, we used splenocytes to characterize the cellular immune response to all seven mRNA vaccines. Groups of BALB/c mice (n = 6) were immunized with two doses (days 0 and 14) of each vaccine (5 μg/mouse for each dose) or empty LNP. To better capture cellular immune responses, splenocytes for evaluating cellular immunogenicity from each vaccinated and control group were collected seven days post the second immunization (days 21) instead of 14 days, as specific T cell responses occur prior to specific antibodies during immune responses [23] (Fig. 4A). Splenocytes from mice in each vaccinated group were re-stimulated with four different types of peptide pools (one pool of overlapping peptides for each RBD of prototype, Delta, Beta, or BA.1), and the resulting cellular immune responses were examined by ICS assay and ELISpot assay.

ICS assay results showed that compared to the LNP group, the CD8^+^ T cells from the splenocytes of all seven vaccinated groups displayed significantly increased percentages of IFN-γ^+^ population in response to re-stimulations comparing to the LNP group (Fig. 4B). ELISpot assay also demonstrated consistent observation that all seven vaccines elicited robust intracellular IFN-γ, with two orders of magnitude increase compared to that in the LNP group (Fig. 4C). These data indicate that all seven vaccines were effective in inducing CD8^+^ T cell responses. Moreover, all vaccinated groups substantially increased the percentage of CD4^+^ cells with intracellular IFN-γ (Fig. 4B). This result suggested that the CD4^+^ T cell response elicited by the seven vaccines was T_H_1-dominant, as IFN-γ is a prototypic T_H_1 cytokine. However, unlike the humoral immune response, CD8^+^ T cells showed diverse responses to different peptide pools after re-stimulation (Fig. 4B). Some vaccinated groups demonstrated a more robust response to re-stimulation by the peptide pool, which was consistent with their immunogens, but others did not. For example, in CD8^+^ splenocytes from the BB group, re-stimulation with the Beta RBD peptide pool induced a higher percentage of IFN-γ^+^ cells than those of other types of re-stimulations. Similarly, CD8^+^ splenocytes from the DO group showed a more robust response to re-stimulation by Delta and BA.1 RBD peptide pools. In addition, CD8^+^ splenocytes from the DD group displayed the most robust response to re-stimulation by prototype RBD peptide pool, while the PP, PB, DB, and OO groups displayed similar levels of cellular immune responses to re-stimulations of all four peptide pools (Fig. 4B). CD4^+^ splenocytes from all vaccinated groups showed no clear association between the cellular immune response to re-stimulation and the constituent RBDs (Fig. 4B). These data indicated that all seven mRNA vaccines could induce cellular immune responses.

### 3.4 Evaluation of chimeric mRNA vaccines as boosters

**Fig. 5:**
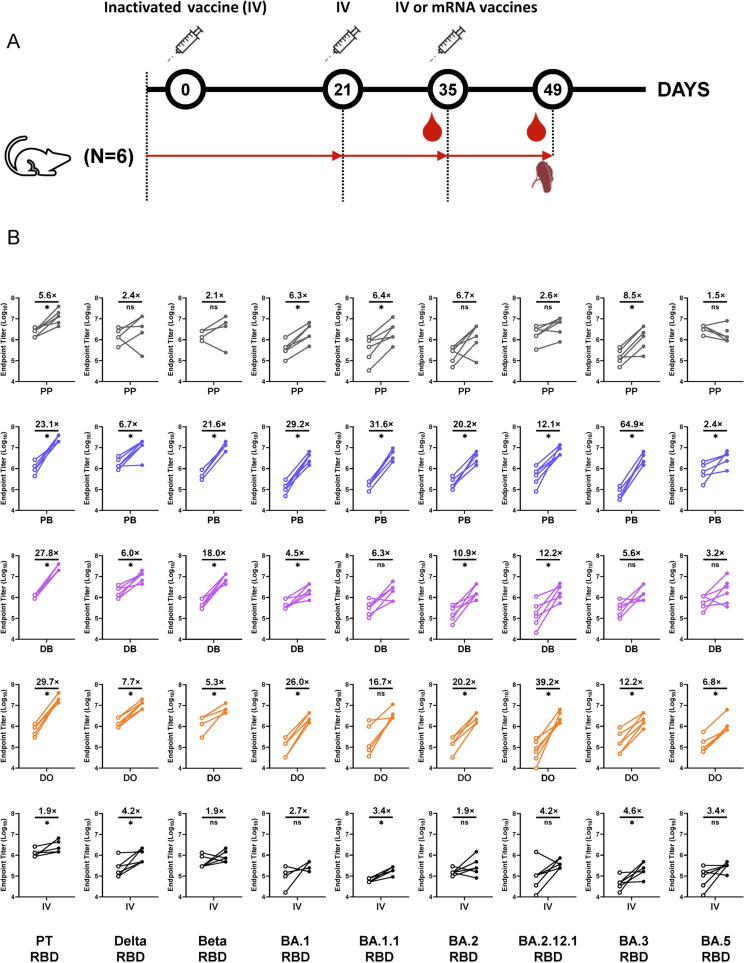
Improvement of IgG titers by three chimeric mRNA vaccines as boosters. A) Mice immunization and sample collection schedule. B) Titers of IgG specific to the receptor-binding domain (RBD) of indicated severe acute respiratory syndrome coronavirus 2 (SARS-CoV-2) variants before (left) and after (right) the booster immunization with each chimeric mRNA vaccine or inactivated vaccine. Numbers indicate geometric mean titer (GMT) fold increase after booster shot. All data are shown as GMT with a 95% confidence interval (CI). Statistical significances were calculated by Wilcoxon matched-pairs signed rank test (*, *P* < 0.05).

Based on the evaluations of humoral and cellular immunogenicity of the seven mRNA vaccines, we chose three mRNA vaccines (PB, DB, and DO) with chimeric RBDs for booster vaccination to provide optimal broad-spectrum immunogenicity. First, the PP vaccine was chosen as a control. Then, we designed a prime-boost experiment to assess the efficacy of these mRNA vaccines as booster vaccinations alongside the same vaccine following two doses of inactivated vaccine. Groups of BALB/c mice (n = 6) were immunized with two doses (days 0 and 21) of inactivated vaccines (2.6 U/mouse each dose) or aluminum adjuvants (Al) only, followed by booster vaccination with the PP, PB, DB, and DO mRNA vaccines (10 μg/mouse) or inactivated vaccines (2.6 U/mouse) on day 35. The control group injected with two doses of Al was further injected with empty LNP as the negative control for the mRNA vaccines. Serum samples were collected 14 days post the second and third doses (days 35 and 49), while splenocytes were collected on day 49 (Fig. 5A).

**Fig. 6:**
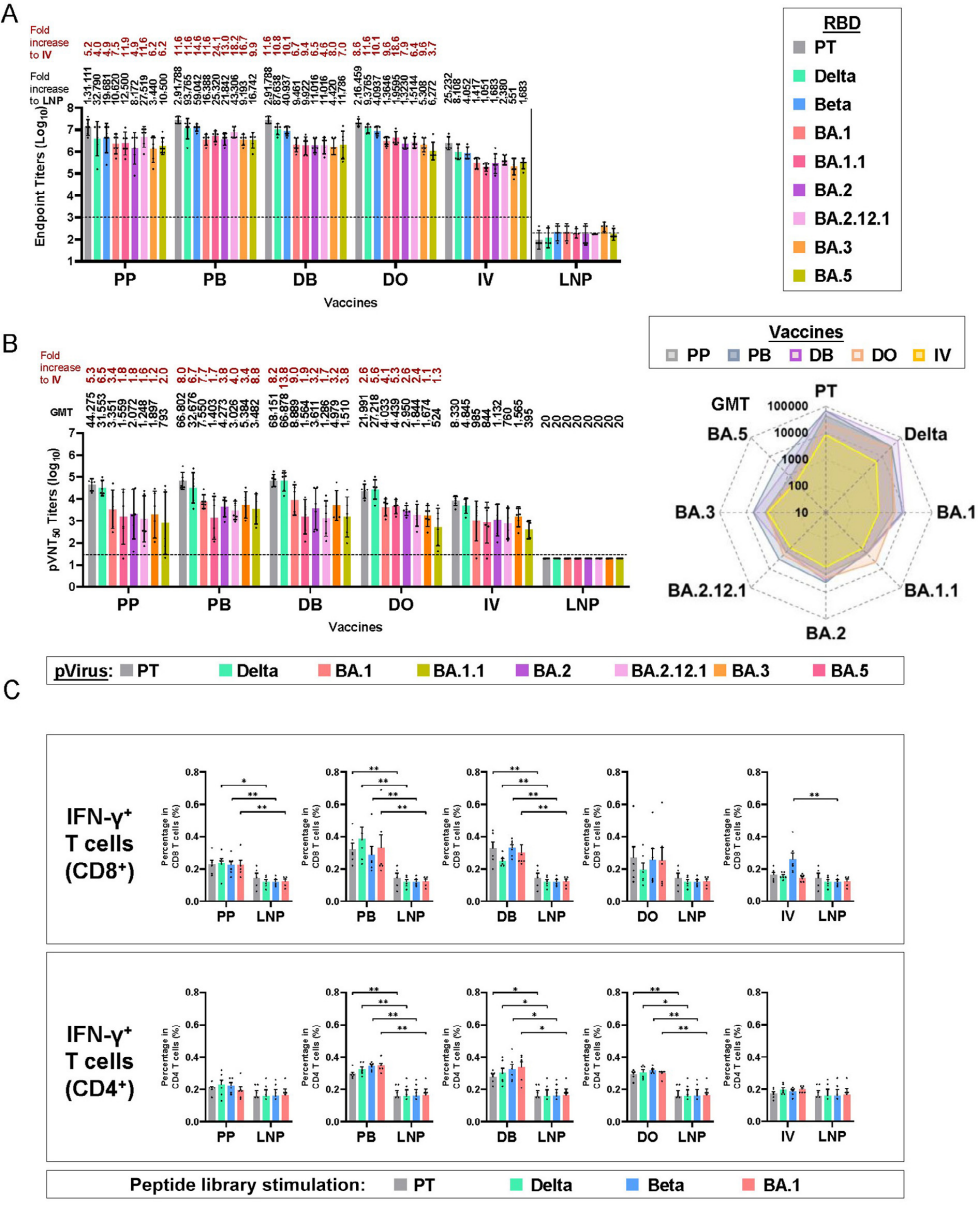
Immunogenicity of the mRNA vaccines with chimeric receptor-binding domains (RBDs) as boosters. A) Titers of IgG specific to the RBD of the indicated severe acute respiratory syndrome coronavirus 2 (SARS-CoV-2) variants after the booster immunization with the indicated mRNA vaccines or IV vaccines. Data are shown as geometric mean titer (GMT) ± 95% confidence interval (CI). The numbers on top indicate the fold increase of IgG titers relative to the cognate RBDs in the lipid nanoparticle (LNP) group or inactivated vaccine (IV) group. B) NT_50_ of neutralizing antibody against the pseudotyped viruses (pVirus) of the indicated SARS-CoV-2 variants (left). The numbers on top indicate GMT and the fold increase of GMT relative to IV. Data are shown as GMT ± 95% CI. A radar map was drawn based on indicated GMT (right). C) Intracellular cytokine staining (ICS) assays quantifying the proportions of IFNγ-secreting CD8^+^ (top) and CD4^+^ (bottom) T cells re-stimulated by the indicated peptide pool. Data are shown as means ± SEM (standard error of the mean). Statistical significances were calculated by Mann-Whitney test (*, *P* < 0.05; **, *P* < 0.01).

First, we examined the humoral immunogenicity of the vaccines as boosters in the serum samples. The titers and binding spectra of IgG elicited by each vaccine specific to the RBDs of nine SARS-CoV-2 variants (prototype, Delta, Beta, BA.1, BA.1.1, BA.2, BA.2.12.1, BA.3 and BA.5) were examined by ELISA (Fig. 5B). Our results showed that a third dose of the vaccine was effective in boosting antibody titers, regardless of the type of vaccine. In addition, booster vaccination with each vaccine significantly increased IgG titers in the serum collected on day 49 (Fig. 5B). However, some vaccines did make a more significant impact than others. For example, compared with inactivated vaccine, the mRNA vaccines (PP, PB, DB, and DO) elicited a 3.7- to 24.1-fold increase of corresponding RBD-specific IgG titers after boost vaccination, indicating they were more potent for boosting IgG levels than the inactivated vaccine (Fig. 6A).

Next, we examined the level of neutralizing antibodies elicited by each boost vaccine against eight pseudotyped viruses of SARS-CoV-2 variants: prototype, Delta, BA.1, BA.1.1, BA.2, BA.2.12.1, BA.3, and BA.5. The serum NT_50_ of neutralization assays showed that when after boost vaccination, the serum from each group contained high levels of neutralizing antibodies, with the highest GMT up to 66802 (Fig. 6B and S4). The neutralizing antibodies elicited by all mRNA vaccines were up to 13.8 folds higher than that elicited by the inactivated vaccine and displayed similar spectra of neutralization that were broader than that of inactivated vaccine. Surprisingly, PB elicited the highest level of neutralizing antibody against BA.5 among all vaccines (Fig. 6B). These data indicate that these mRNA vaccines were more effective than inactivated vaccines as booster shots.

Second, we examined the cellular immunogenicity of these vaccines after boost vaccination. Splenocytes from each sample were re-stimulated with the peptide pools of RBDs from the prototypes Delta, Beta, or BA.1, and the percentages of IFN-γ^+^ T cells in the CD8^+^ or CD4^+^ population were examined by flow cytometry. Our data showed that PP, PB, and DB elicited a significantly increased percentage of IFN-γ^+^ population CD8^+^ T cells compared with LNP after re-stimulation by the RBD peptide pool of Delta, Beta, or BA.1 (Fig. 6C). DO-elicited CD8^+^ T cells also showed a slightly higher response than LNP to re-stimulation. Meanwhile, PB, DB, and DO elicit significant CD4^+^ T cells response to all four types of re-stimulation, but PP or inactivated vaccine did not. Together, these data demonstrate that, with one dose of mRNA vaccine booster following inactivated vaccines, the cellular immune response can be potently elicited.

## 4 Discussion

The persistent emergence and circulation of SARS-CoV-2 variants raised the challenge for the COVID-19 pandemic control. The current Omicron VOC showed the most severe resistance to the immune responses induced by early SARS-CoV-2 strains infection or COVID-19 vaccines designed with the prototype virus [24], [25]. Therefore, it is crucial to develop next-generation vaccines with broad-spectrum immunogenicity. In addition, considering the high COVID-19 vaccine inoculation coverage in most countries, the immunogenicity induced by the vaccine candidates as booster shots should be focused on closely.

In many countries, first-generation COVID-19 vaccines have been administered as booster shots, such as the inactivated vaccine BBIBP-CoV, protein subunit vaccine ZF2001®, mRNA vaccines mRNA-1273 and BNT162b2. However, the severe immune escape of Omicron sub-variants urges the update of vaccines. Recently, Moderna and Pfizer-BioNTech updated their mRNA vaccines with Omicron immunogens and formulated bivalent (prototype + Omicron) vaccines that displayed potent efficacy to Omicron sub-variants in clinical trials [26]. As bivalent booster shots, the Moderna and Pfizer-BioNTech vaccines have been approved in over 30 countries, including the United States, the United Kingdom, Canada, Japan, and European Medicine Agency (EMA) countries. In addition, GSK/Sanofi also developed a protein subunit booster vaccine with a Beta variant immunogen (MVB.1.351) that displayed effective neutralization against Omicron in clinical trials [27].

Here, we provide a series of mRNA vaccine candidates for boost vaccination. These vaccines induce broad-spectrum immunogenicity to a wide range of SARS-CoV-2 mutant strains, especially the currently circulating Omicron BA.5 sub-variant. In our previous work, we developed the COVID-19 protein subunit vaccine ZF2001® based on the tandem repeat RBD-dimer [11], the chimeric RBD-dimers protein vaccines with broad-spectrum immunogenicity against SARS-CoV-2 variants [18]. In addition, we verified the feasibility of using homotypic and chimeric RBD-dimers as the immunogens of mRNA vaccines [19]. In this work, we first evaluated the immunogenicity of a panel of seven dimeric RBD mRNA vaccines of homologous or heterologous RBDs in naïve mice to multiple Omicron sub-variants (BA.1, BA.1.1, BA.2, BA.2.12.1, BA.3 and BA.5) and three other variants (prototype, Delta, and Beta). In addition, we identified DO, among seven vaccines, as the most broad-spectrum candidate. With these data, we chose the chimeric mRNA vaccine candidates, PB, DB, and DO, as booster shots following two doses of the inactivated vaccines. We found that these vaccines elicited superior broad-spectrum immunogenicity against prototype, Delta, and Omicron sub-variants, including BA.5 than that elicited by inactivated vaccines, which makes them suitable to serve as booster shots in countries primarily vaccinated with two doses of inactivated vaccines.

Interestingly, as a booster shot, PB, instead of DO, stimulated the most potent immune response to BA.5, unlike the immune response of naïve mice administered with two doses of mRNA vaccines. The effectiveness of the PB vaccine against Omicron sub-variants is consistent with other Beta-containing vaccines such as MVB.1.351 [27]. Moreover, a previous report discovered that BA.1 breakthrough infection mainly recalled memory B cells induced by SARS-CoV-2 prototype antigen [28], suggesting that Omicron-containing vaccines may tend to elicit prototype-specific neutralizing antibodies instead of Omicron-specific ones with prior vaccination of prototype-based vaccines. In addition, Beta variant infection elicited potent both Beta-specific and cross-reactive antibodies [29]. As prototype-specific antibodies display poor neutralization activity against Omicron, this may explain why PB outperformed DO as booster shots. Thus, further investigations are needed to dissect the underlying mechanisms.

In addition, mice vaccinated with two doses of DD displayed higher T cell responses than other vaccines (Fig. 4B and S5). Using the NetMHC-4.0 website, an L452R-containing peptide (NYNYRYRLF) in Delta RBD displays twice as high binding affinity as its corresponding peptide (NYNYLYRLF) in prototype RBD (Table S1), which probably leads to the better cellular immunogenicity of L452R-containing mRNA vaccines, especially DD that contains two copies of Delta RBDs with tandem repeat.

In conclusion, this study evaluated the immunogenicity of chimeric RBD mRNA vaccines against multiple Omicron sub-variants and their performance as booster shots following two doses of inactivated vaccines. With these data, we discovered promising broad-spectrum vaccine candidates that could serve as the third dose for booster vaccination. We believe these vaccines will significantly contribute to the global fight against COVID-19.

## Ethics statement

This study was carried out following the recommendations described in the Guide for the Care and Use of Laboratory Animals of the Institute of Microbiology, Chinese Academy of Sciences (IMCAS) Ethics Committee. Furthermore, all animal experiments were reviewed and approved by the Committee on the Ethics of Animal Experiments of IMCAS (No. APIMCAS2022026).
