# Supplementary material for: Rapid evaluation of heterologous chimeric RBD-dimer mRNA vaccine for currently-epidemic Omicron sub-variants as booster shot after inactivated vaccine
Source: Biosaf Health. 2023 Mar 2;5(2):89–100. doi: 10.1016/j.bsheal.2023.02.002 (PMC9979697; doi:10.1016/j.bsheal.2023.02.002)
Supplement: Supplementary data 1 [file mmc1.pdf]

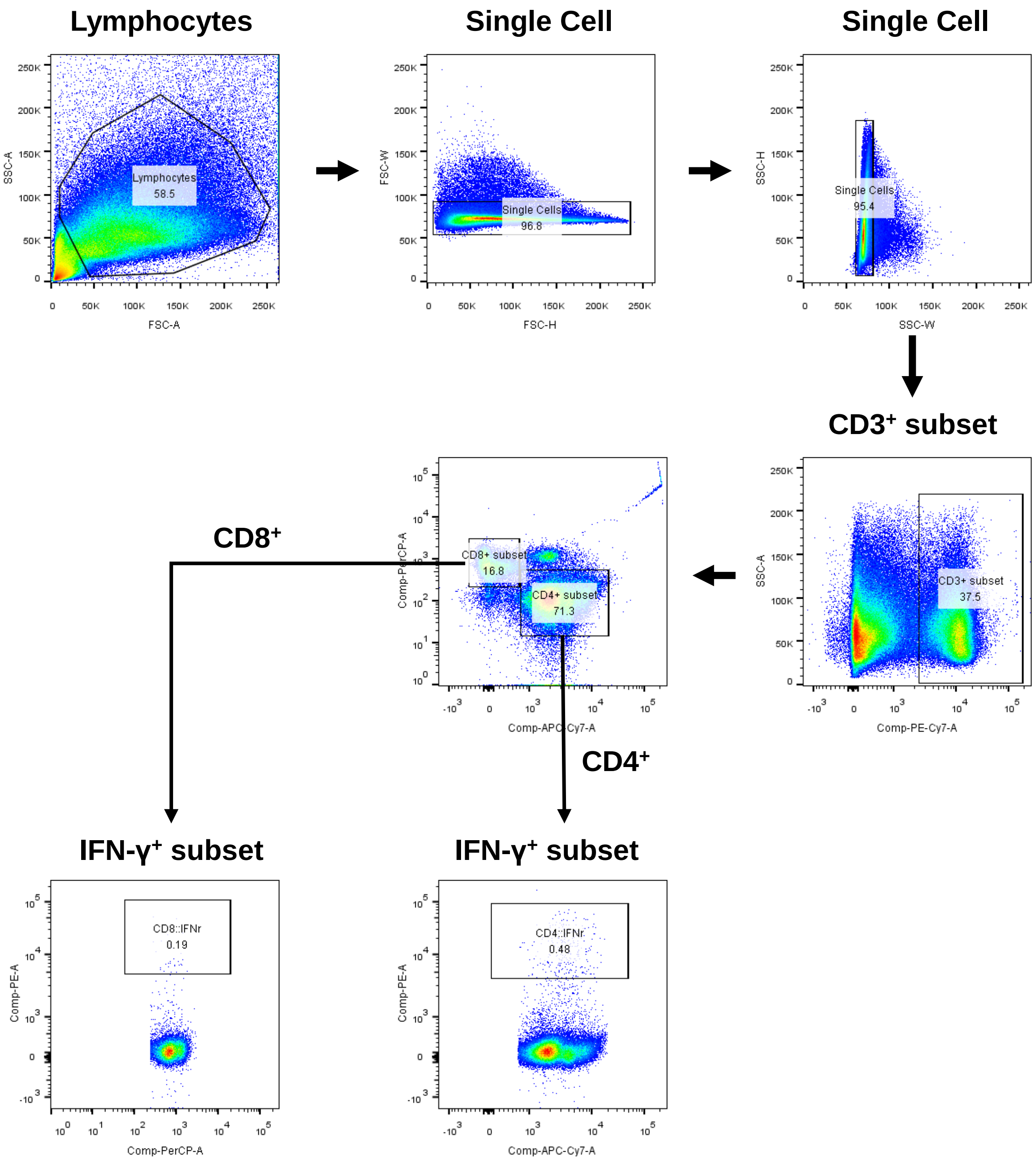

Figure S1. Gating strategy of ICS assay.

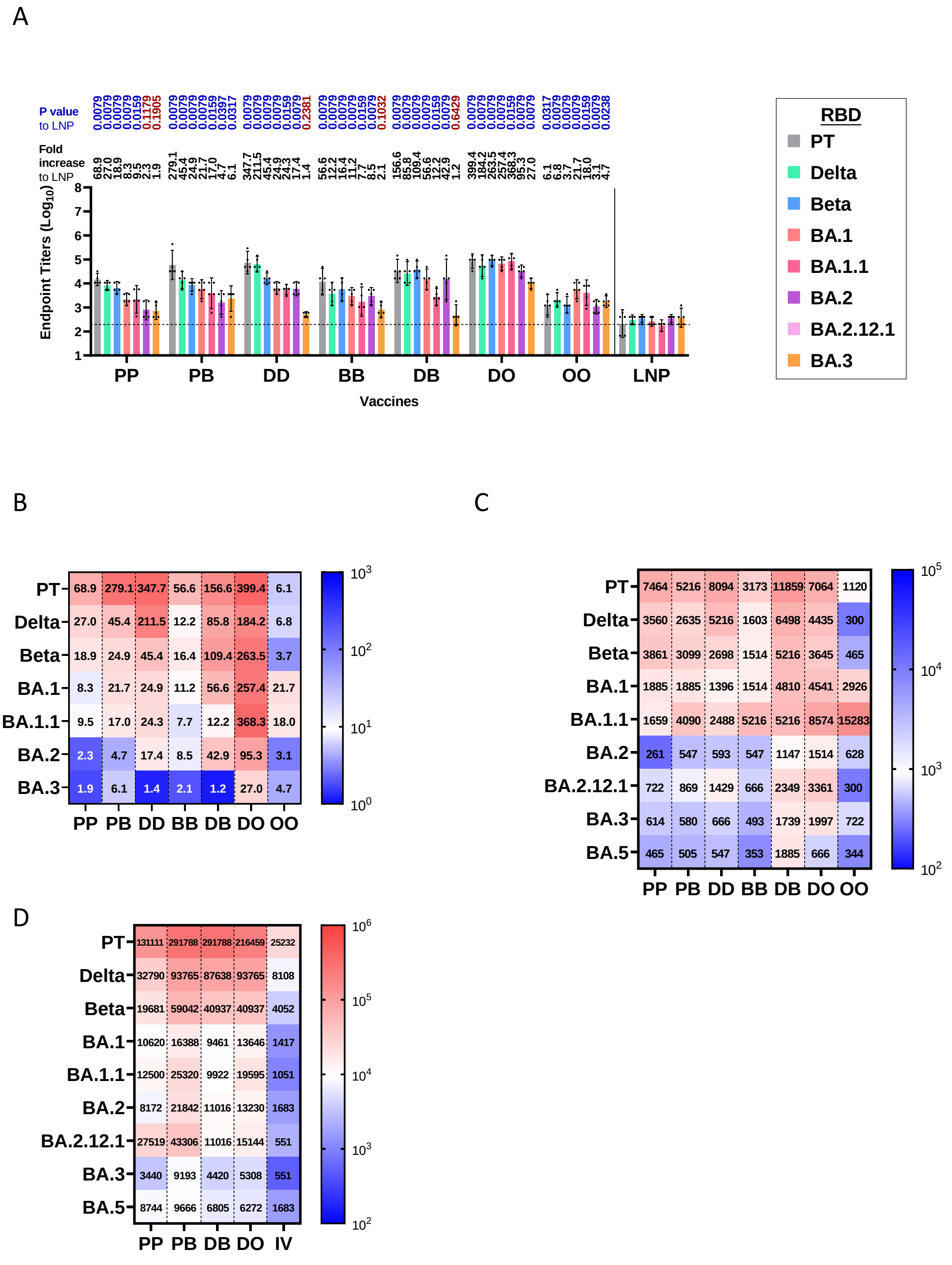

Figure S2. Evaluation of humoral immunogenicity of mRNA vaccines with dimeric RBD of SARS-CoV-2. A) Titers of IgG specific to the RBD of the indicated SARS-CoV-2 variants at day 14. Numbers on top indicate the fold-increase or the p-value relative to the cognate RBDs in the LNP group. Blue and red letters indicate statistically significant or not significant p-values, respectively. P-values were calculated by Mann-Whitney test. B and C) Heatmaps were drawn based on the fold increase of the titers of IgG specific to the RBD of SARS-CoV-2 variants at day 14 (B) and day 28 (C), comparing the cognate RBDs in the LNP group. D) Heatmaps of the evaluation of humoral immunogenicity of the chimeric mRNA vaccines as boosters drawn based on the fold increase of the titers of IgG specific to the RBD of SARS-CoV-2 variants comparing the cognate RBDs in the LNP group.

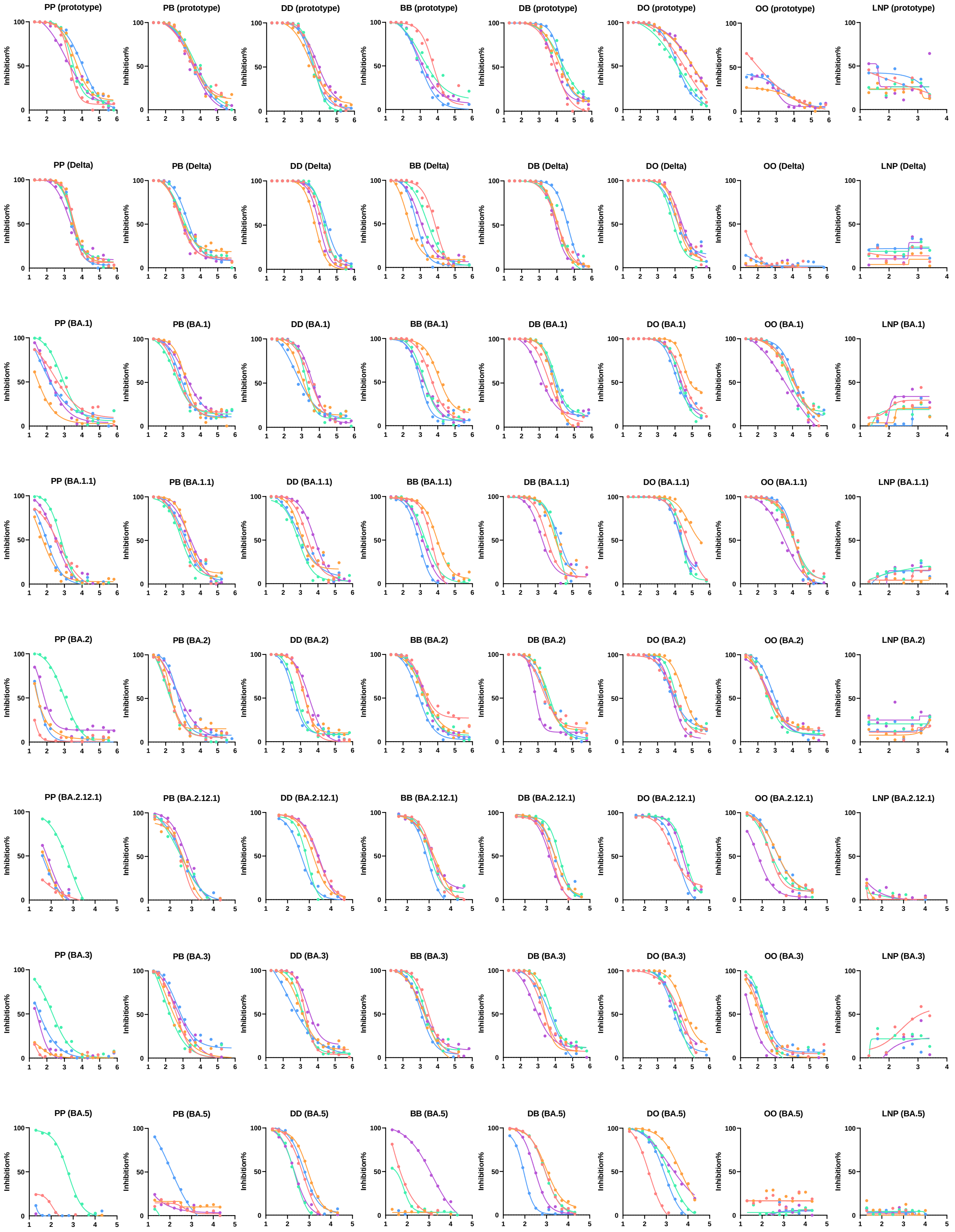

Figure S3. Neutralization curves of antibodies elicited by seven dimeric mRNA vaccines against eight pseudotyped viruses of SARS-CoV-2 variants. Numbers on X axis indicate dilution (log_{10}). Lines in the same graph represent neutralization by serum samples from the same group.

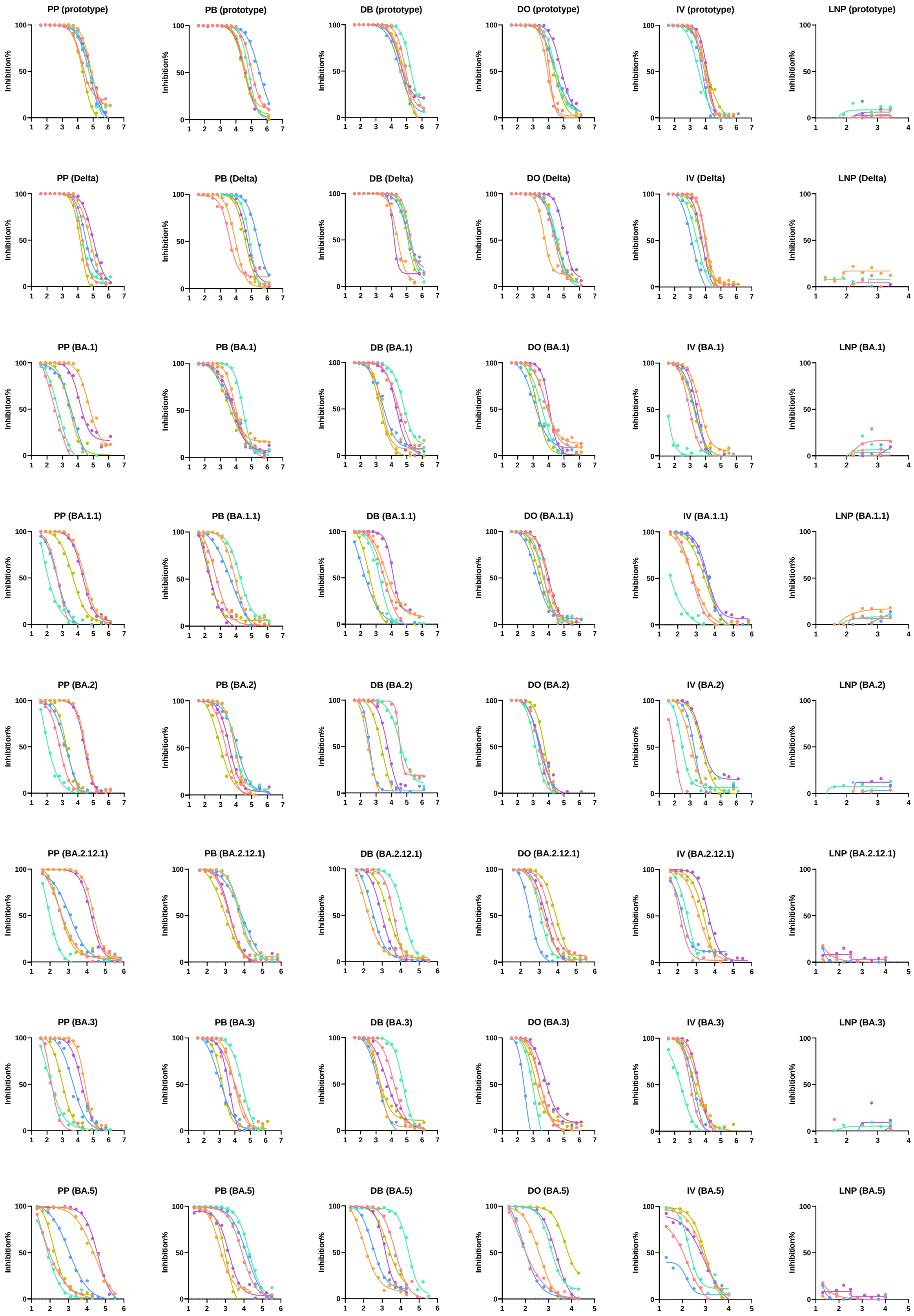

Figure S4. Neutralization curves of antibodies elicited by chimeric mRNA vaccines as boosters against eight pseudotyped viruses of SARS-CoV-2 variants. Numbers on X axis indicate dilution ($\log_{10}$). Lines in the same graph represent neutralization by serum samples from the same group.

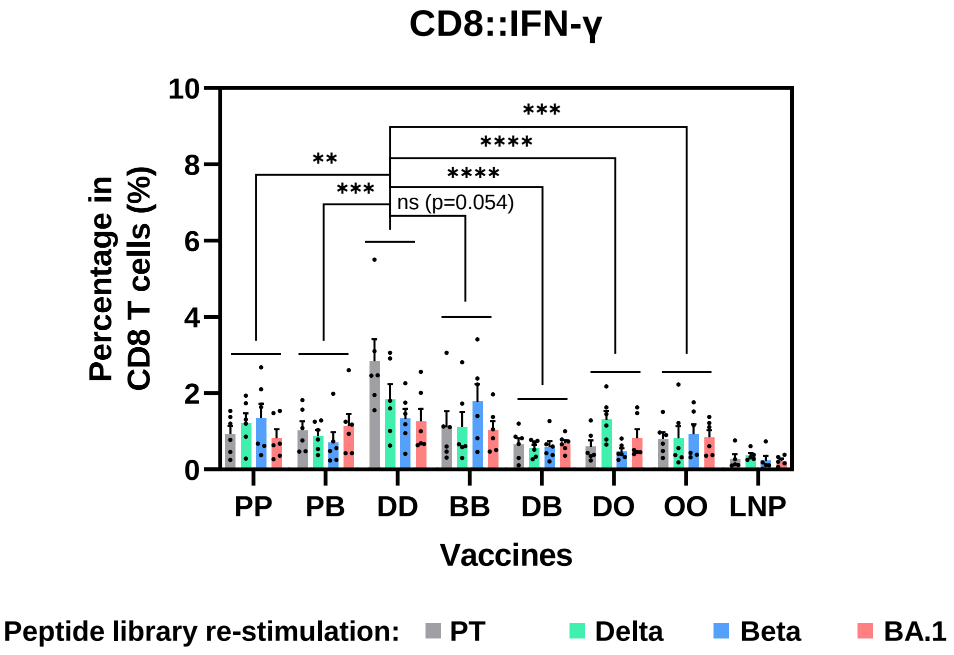

Figure S5. Increased CD8⁺ T cells response in mice vaccinated with two doses of DD. ICS assays quantifying the proportions of IFNγ-secreting CD8⁺ T cells stimulated by the indicated peptide pool. Data are shown as means ± SEM. Statistical significances were calculated by Ordinary two-way ANOVA. P values represent the variance of different vaccination groups (*, p<0.05; **, p<0.01; ***, p<0.001; ****, p<0.0001).

HLA	peptide	Affinity(nM)	%Rank BindLevel
H-2-Dd	NYNY**L**YRLF	25844.92	7.50
H-2-Dd	NYNY**R**YRLF	12378.86	1.50 <= WB
H-2-Kd	NYNY**L**YRLF	12286.93	7.00
H-2-Kd	NYNY**R**YRLF	17938.45	12.00

Table S1. Binding affinity of 9-mer peptide without or with L452R mutation. Predication of binding affinity to MHC class I was conducted using NetMHC-4.0 (https://services.healthtech.dtu.dk/service.php?NetMHC-4.0). The alleles of HLA was set as H-2-Dd and H-2-Kd for BALB/c mice. **Bold** letters indicate L452 or R452. WB represents weak binding (Rank Threshold 2.00 %).
